# Supplementary material for: The role of maternal preconception vitamin D status in human offspring sex ratio
Source: Nat Commun. 2021 May 13;12:2789. doi: 10.1038/s41467-021-23083-2 (PMC8119683; doi:10.1038/s41467-021-23083-2)
Supplement: Supplementary file 1 — Supplementary Information [file 41467_2021_23083_MOESM1_ESM.pdf]

Supplementary Table 1. Adjusted risk differences (RDs) and 95% confidence intervals (CIs) for 25-hydroxyvitamin D [25(OH)D] and male live birth, Effects of Aspirin in Gestation and Reproduction (EAGeR) trial, 2007-2011[a,b]

		Among complete follow-up (n=1,094)	Among pregnancies (n=803)[c]	Among live births (n=601)[d]
	N[e]	Adjusted[f] RD (95% CI)	Adjusted[f] RD (95% CI)	Adjusted[f] RD (95% CI)
25(OH)D				
<30 ng mL^{-1} (referent)	135	1	1	1
≥30 ng mL^{-1}	157	5.60 (0.35, 10.85)	4.05 (-2.76, 10.85)	3.63 (-4.45, 11.76)
Among hsCRP≤1.95 ng mL^{-1}				
<30 ng mL^{-1} (referent)	92	1	1	1
≥30 ng mL^{-1}	118	2.60 (-3.92, 9.12)	0.69 (-7.63, 9.02)	0.12 (-9.75, 9.98)
Among hsCRP>1.95 ng mL^{-1}				
<30 ng mL^{-1} (referent)	43	1	1	1
≥30 ng mL^{-1}	39	7.88 (-0.36, 16.11)	7.60 (-6.91, 22.12)	8.10 (-11.92, 28.11)

[a]8 twin gestations contributed two observations each to the analysis.

[b]RDs (per 100) and 95% CIs calculated using multiply-imputed generalized estimating equations of log-binomial regression with robust standard errors and stabilized inverse-probability weights to account for loss to follow-up. Poisson models used in instances of model non-convergence.

[c]Models further weighted to account for selection of pregnancies.

[d]Models further weighted to account for selection of pregnancies and survival to live birth.

[e]Number of live-born males.

[f]Multivariable model adjusted for age (continuous), race/ethnicity (white or non-white), number of previous live births (0, 1, or ≥2).

Supplementary Table 2. Adjusted relative risks (RRs) and 95% confidence intervals (CIs) for 25-hydroxyvitamin D [25(OH)D] and female live birth, Effects of Aspirin in Gestation and Reproduction (EAGeR) trial, 2007-2011[a]

	N[d]	RR (95% CI)[b,c] Among complete follow-up (n=1,094)	Among pregnancies (n=803)[e]	Among live births (n=601)[f]
25(OH)D				
per 10 ng mL^{-1}	309	1.07 (1.00, 1.15)	1.04 (0.97, 1.12)	0.97 (0.89, 1.06)
<30 ng mL^{-1} (referent)	158	1	1	1
≥30 ng mL^{-1}	151	1.05 (0.87, 1.28)	0.96 (0.79, 1.16)	0.86 (0.71, 1.06)
Among hsCRP≤1.95 ng mL^{-1}				
<30 ng mL^{-1} (referent)	93	1	1	1
≥30 ng mL^{-1}	112	1.08 (0.85, 1.37)	1.03 (0.82, 1.30)	0.99 (0.78, 1.24)
Among hsCRP>1.95 ng mL^{-1}				
<30 ng mL^{-1} (referent)	65	1	1	1
≥30 ng mL^{-1}	39	0.99 (0.70, 1.40)	0.90 (0.63, 1.28)	0.86 (0.65, 1.15)

[a]8 twin gestations contributed two observations to the analysis.
[b]RRs, RDs (per 100), and 95% CIs calculated using multiply-imputed generalized estimating equations of log-binomial regression with robust standard errors with stabilized inverse-probability weights to account for loss to follow-up.
[c]Multivariable model adjusted for age (continuous), race/ethnicity (white or non-white), number of previous live births (0, 1, or ≥2).
[d]Number of live-born females.
[e]Models additionally weighted for selection of pregnancies.
[f]Models further weighted to account for selection of pregnancies and survival to live birth.

Supplementary Table 3. Adjusted relative risks (RRs), risk differences (RDs), and 95% confidence intervals (CIs) for 25-hydroxyvitamin D [25(OH)D] and pregnancy with a female or male fetus among 954 women and mother-offspring pairs, with either a pregnancy with offspring known or completion of follow-up without pregnancy, Effects of Aspirin in Gestation and Reproduction (EAGeR) trial, 2007-2011[a-c]

		Pregnancy with Female Fetus			Pregnancy with Male Fetus	
	N[d]	RR (95% CI)	RD (95% CI)	N[e]	RR (95% CI)	RD (95% CI)
25(OH)D						
<30 ng mL^{-1} (referent)	173	1	1	146	1	1
≥30 ng mL^{-1}	171	1.06 (0.89, 1.26)	2.39 (-3.78, 8.57)	167	1.22 (1.01, 1.47)	5.98 (-0.02, 11.93)
Among hsCRP≤1.95 ng mL^{-1}						
<30 ng mL^{-1} (referent)	101	1	1	99	1	1
≥30 ng mL^{-1}	125	1.07 (0.86, 1.33)	2.71 (-4.92, 10.33)	128	1.11 (0.90, 1.38)	3.25 (-4.20, 10.71)
Among hsCRP>1.95 ng mL^{-1}						
<30 ng mL^{-1} (referent)	72	1	1	47	1	1
≥30 ng mL^{-1}	46	1.06 (0.78, 1.43)	2.18 (-8.82, 13.19)	39	1.34 (0.93, 1.92)	7.20 (-2.24, 16.84)

[a]8 twin gestations contributed two observations to the analysis.

[b]RRs, RDs (per 100), and 95% CIs calculated using multiply-imputed generalized estimating equations of log-binomial regression with robust standard errors with stabilized inverse-probability weights to account for loss to follow-up.

[c]Multivariable model 3 adjusted for age (continuous), race/ethnicity (white or non-white), number of previous live births (0, 1, or ≥2).

[d]Number of female pregnancies.

[e]Number of male pregnancies.

Supplemental Table 4. Preconception 25-hydroxyvitamin D [25(OH)D] status in relation to male offspring among 56 pregnancy losses with sex determined by genetic analysis, Effects of Aspirin in Gestation and Reproduction (EAGeR) trial, 2007-2011[a]

	All pregnancy losses		Euploid losses		Aneuploid losses	
	<30 ng mL^{-1}	≥30 ng mL^{-1}	<30 ng mL^{-1}	≥30 ng mL^{-1}	<30 ng mL^{-1}	≥30 ng mL^{-1}
Original						
Male	10	11	6	3	4	7
Female	15	20	9	8	6	12
Total	25	31	15	11	10	19
OR (95% CI)[b]	0.83 (0.50, 1.62)		0.56 (0.10, 3.02)		0.88 (0.18, 4.21)	
Revised[c]						
Male	12	14	7	4	5	9
Female	13	17	6	9	7	8
Total	25	31	13	13	12	17
OR (95% CI)[b]	0.89 (0.31, 2.56)		0.38 (0.08, 1.90)		1.58 (0.36, 7.00)	

[a]2 pregnancy losses were twin gestations: one male-male pair and one female-female pair. No genetic analysis available for one phenotypic male, who was excluded from stratified analysis.

[b]Odds ratios (ORs) and 95% confidence intervals (CIs) for male pregnancy loss associated with sufficient versus insufficient preconception vitamin D status among losses with available karyotype data.

[c]Distributions of sex and euploid/aneuploid status were revised according to a validation study.

Supplementary Table 5. Adjusted relative risks (RRs) and 95% confidence intervals (CIs) for 25-hydroxyvitamin D [25(OH)D] and male live birth, stratified by eligibility criteria, Effects of Aspirin in Gestation and Reproduction (EAGeR) trial, 2007-2011[a,b]

	N[e]	Among complete follow-up (n=1,094) Adjusted[f] RR (95% CI)	Among pregnancies (n=803)[c] Adjusted[f] RR (95% CI)	Among live births (n=601)[d] Adjusted[f] RR (95% CI)
Original stratum				
<30 ng mL^{-1} (referent)	60	1	1	1
≥30 ng mL^{-1}	89	1.48 (1.12, 1.96)	1.29 (0.98, 1.69)	1.18 (0.91, 1.53)
Expanded stratum				
<30 ng mL^{-1} (referent)	90	1	1	1
≥30 ng mL^{-1}	81	1.06 (0.79, 1.41)	0.99 (0.73, 1.33)	1.01 (0.74, 1.42)

[a]8 twin gestations contributed two observations each to the analysis.

[b]RRs and 95% CIs calculated using multiply-imputed generalized estimating equations of log-binomial regression with robust standard errors and stabilized inverse-probability weights to account for loss to follow-up. Poisson models used in instances of model non-convergence.

[c]Models further weighted to account for selection of pregnancies.

[d]Models further weighted to account for selection of pregnancies and survival to live birth.

[e]Number of live-born males.

[f]Multivariable model adjusted for age (continuous), race/ethnicity (white or non-white), number of previous live births (0, 1, or ≥2).
