# Supplementary material for: The role of maternal preconception vitamin D status in human offspring sex ratio
Source: Nat Commun. 2021 May 13;12:2789. doi: 10.1038/s41467-021-23083-2 (PMC8119683; doi:10.1038/s41467-021-23083-2)
Supplement: Supplementary file 3 — Reporting Summary [file 41467_2021_23083_MOESM3_ESM.pdf]

nature research

Corresponding author(s): Dr. Sunni Mumford

Last updated by author(s): Jan 13, 2021

Reporting Summary

Nature Research wishes to improve the reproducibility of the work that we publish. This form provides structure for consistency and transparency in reporting. For further information on Nature Research policies, see our Editorial Policies and the Editorial Policy Checklist.

Statistics

For all statistical analyses, confirm that the following items are present in the figure legend, table legend, main text, or Methods section.

n/a	Confirmed	
☐	☒	The exact sample size (n) for each experimental group/condition, given as a discrete number and unit of measurement
☒	☐	A statement on whether measurements were taken from distinct samples or whether the same sample was measured repeatedly
☐	☒	The statistical test(s) used AND whether they are one- or two-sided<br>*Only common tests should be described solely by name; describe more complex techniques in the Methods section.*
☐	☒	A description of all covariates tested
☐	☒	A description of any assumptions or corrections, such as tests of normality and adjustment for multiple comparisons
☐	☒	A full description of the statistical parameters including central tendency (e.g. means) or other basic estimates (e.g. regression coefficient) AND variation (e.g. standard deviation) or associated estimates of uncertainty (e.g. confidence intervals)
☒	☐	For null hypothesis testing, the test statistic (e.g. F, t, r) with confidence intervals, effect sizes, degrees of freedom and P value noted<br>*Give P values as exact values whenever suitable.*
☒	☐	For Bayesian analysis, information on the choice of priors and Markov chain Monte Carlo settings
☒	☐	For hierarchical and complex designs, identification of the appropriate level for tests and full reporting of outcomes
☒	☐	Estimates of effect sizes (e.g. Cohen's d, Pearson's r), indicating how they were calculated

Our web collection on statistics for biologists contains articles on many of the points above.

Software and code

Policy information about availability of computer code

Data collection	No software was used for data collection.
Data analysis	All analyses were conducted using SAS v9.4 software (Cary, NC) and R-4.0.3. The code used to generate the results that appear in the manuscript can be found on GitHub (https://github.com/apurduesmithe/vitdsexratio).

For manuscripts utilizing custom algorithms or software that are central to the research but not yet described in published literature, software must be made available to editors and reviewers. We strongly encourage code deposition in a community repository (e.g. GitHub). See the Nature Research guidelines for submitting code & software for further information.

Data

Policy information about availability of data

All manuscripts must include a data availability statement. This statement should provide the following information, where applicable:

- Accession codes, unique identifiers, or web links for publicly available datasets
- A list of figures that have associated raw data
- A description of any restrictions on data availability

The data that support the findings of this study are available from the corresponding author upon reasonable request.

Field-specific reporting

Please select the one below that is the best fit for your research. If you are not sure, read the appropriate sections before making your selection.

☒ Life sciences ☐ Behavioural & social sciences ☐ Ecological, evolutionary & environmental sciences

For a reference copy of the document with all sections, see nature.com/documents/nr-reporting-summary-flat.pdf

Life sciences study design

All studies must disclose on these points even when the disclosure is negative.

Sample size	This was a secondary analysis of the EAGeR trial; therefore, the sample size for our analyses was predetermined. Sample size calculations for the original aims of the EAGeR trial (n=1,228) have been described at length elsewhere. (Schisterman et al., 2014) Briefly, the study was initially designed to detect a 10% absolute difference in live birth rate with 80% power and a 5% type I error rate, assuming participants taking placebo who achieved pregnancy had a 75% live birth rate. Therefore, a sample size of 1254 was needed assuming a 40% pregnancy rate over six months of trying. Accounting for a potential 20% loss to follow-up, 1600 was the recruitment target. Additional calculations based on unconditional probabilities of live birth indicated a power of 90% to detect a 10% increase in LDA assuming a 50% live birth rate in the placebo arm, with a sample size of 1076 complete cases. The final overall number recruited was 1228 due to slower than anticipated rate of recruitment and funding constraints. However, the actual loss to follow-up of 12% (n=150) was less than the 20% expected.
Data exclusions	There were no data exclusions.
Replication	The code and analyses were double- and triple-checked by the first author.
Randomization	Given that this was an observational study, vitamin D status was not randomly assigned. However, we took care to control for important confounders in main regression models, including age, race/ethnicity, number of previous live births.
Blinding	Given that the exposure, vitamin D status, was not randomly assigned in this observational study, blinding was not relevant to our study.

Reporting for specific materials, systems and methods

We require information from authors about some types of materials, experimental systems and methods used in many studies. Here, indicate whether each material, system or method listed is relevant to your study. If you are not sure if a list item applies to your research, read the appropriate section before selecting a response.

Materials & experimental systems

n/a	Involved in the study	
☒	☐	Antibodies
☒	☐	Eukaryotic cell lines
☒	☐	Palaeontology and archaeology
☒	☐	Animals and other organisms
☐	☒	Human research participants
☐	☒	Clinical data
☒	☐	Dual use research of concern

Methods

n/a	Involved in the study	
☒	☐	ChIP-seq
☒	☐	Flow cytometry
☒	☐	MRI-based neuroimaging

Human research participants

Policy information about studies involving human research participants

Population characteristics	Study participants included 1,228 women attempting pregnancy, ages 18–40 years, with 1 to 2 prior pregnancy losses. Women with known history of infertility treatment, pelvic inflammatory disease, tubal occlusion, endometriosis, anovulation and polycystic ovary syndrome, or uterine abnormality were ineligible to participate.
Recruitment	Participants were recruited at four clinical sites in the United States from 2007-2011 and followed for up to six menstrual cycles while attempting pregnancy and then throughout pregnancy for women who conceived. Among 1,228 women randomized, 1,088 completed follow up (89%). Given the minimal loss to follow-up, combined with our use of inverse-probability weighting to account for selection bias arising from loss to follow-up, we do not expect selection bias to impact our results in any meaningful way.
Ethics oversight	The institutional review board at each study site (Salt Lake City, Utah; Denver, Colorado; Buffalo, New York; Scranton, Pennsylvania) and data coordinating center approved the trial protocol and all participants provided written informed consent prior to enrolling. The original trial was registered with ClinicalTrials.gov, number NCT00467363.

Note that full information on the approval of the study protocol must also be provided in the manuscript.

Clinical data

Policy information about clinical studies

All manuscripts should comply with the ICMJE guidelines for publication of clinical research and a completed CONSORT checklist must be included with all submissions.

Clinical trial registration	The original trial was registered with ClinicalTrials.gov, number NCT00467363.
Study protocol	The study protocol for the EAGeR trial are available as Supplemental Material.
Data collection	Participants were recruited at four clinical sites in the United States from 2007-2011 and followed for up to six menstrual cycles while attempting pregnancy and then throughout pregnancy for women who conceived.
Outcomes	The primary outcome was live birth. Secondary outcomes included implantation (positive urine pregnancy test (PPT)), confirmed pregnancy (the sonogram showed a gestational sac, clinical documentation of fetal heart tones, or a later stage confirmation of pregnancy), pregnancy loss (<20 weeks' gestation after documentation of confirmed pregnancy), birth weight, and obstetrical complications. Complications included preeclampsia, gestational diabetes, gestational hypertension, and preterm birth (<37 weeks) and were obtained prospectively by maternal report and medical record abstraction by trained staff. Gestational age was based on the six to seven week study sonogram. For participants with a pregnancy loss before the early sonogram, gestational age was defined as the number of days between ovulation detected on the fertility monitor to the date of the loss plus 14 days.
